# The ADAMTS5 Metzincin Regulates Zebrafish Somite Differentiation

**DOI:** 10.3390/ijms19030766

**Published:** 2018-03-07

**Authors:** Carolyn M. Dancevic, Yann Gibert, Joachim Berger, Adam D. Smith, Clifford Liongue, Nicole Stupka, Alister C. Ward, Daniel R. McCulloch

**Affiliations:** 1 School of Medicine, Deakin University, Waurn Ponds, Victoria 3216, Australia; carolyn.dancevic@deakin.edu.au (C.M.D.); y.gibert@deakin.edu.au (Y.G.); adam.smith@bio-strategy.com (A.D.S.); c.liongue@deakin.edu.au (C.L.); nicole.stupka@deakin.edu.au (N.S.); daniel.mcculloch@uq.net.au (D.R.M.); 2 Centre for Molecular and Medical Research, Deakin University, Waurn Ponds, Victoria 3216, Australia; 3 Australian Regenerative Medicine Institute, Monash University, Clayton, Victoria 3800, Australia; joachim.berger@monash.edu

**Keywords:** metalloproteinase, extracellular matrix, ADAMTS, somite, muscle, zebrafish

## Abstract

The ADAMTS5 metzincin, a secreted zinc-dependent metalloproteinase, modulates the extracellular matrix (ECM) during limb morphogenesis and other developmental processes. Here, the role of ADAMTS5 was investigated by knockdown of zebrafish *adamts5* during embryogenesis. This revealed impaired Sonic Hedgehog (Shh) signaling during somite patterning and early myogenesis. Notably, synergistic regulation of *myod* expression by ADAMTS5 and Shh during somite differentiation was observed. These roles were not dependent upon the catalytic activity of ADAMTS5. These data identify a non-enzymatic function for ADAMTS5 in regulating an important cell signaling pathway that impacts on muscle development, with implications for musculoskeletal diseases in which ADAMTS5 and Shh have been associated.

## 1. Introduction

The A Disintegrin-like and Metalloproteinase domain with Thrombospondin-1 motifs (ADAMTS) metalloproteinases have important functions during developmental morphogenesis and are also implicated in chronic disease. The proteoglycanase subfamily of ADAMTS1, 4, 5, 8, 9, 15 and 20 have broad functions, many attributed to their ability to remodel extracellular matrix (ECM) components, such as the chondroitin sulphate proteoglycans versican and aggrecan. For example, *Adamts20* deficient *bt/bt* mice have defects in melanoblast survival [1] and *Adamts9* haplo-insufficient mice on an *Adamts20* deficient (*bt/bt*) background present with a secondary cleft palate [2], in each case associated with reduced versican proteolysis. Furthermore, ADAMTS1 has been implicated in promoting atherosclerosis [3] and ADAMTS15 acts as a tumor suppressor in breast carcinoma [4], potentially through proteoglycan proteolysis. However, non-enzymatic roles for several ADAMTS family members have been described [5,6,7]. 

ADAMTS5 has been implicated in classic morphogenesis during development as well as in chronic diseases such as arthritis and atherosclerosis. For example, combinatorial knockout of *Adamts5*, *Adamts9* and *Adamts20* in mice prevented generation of bioactive fragments of versican that are necessary for interdigital tissue apoptosis during development [8,9]. *Adamts5* knockout mice also developed myxomatous heart valves [10]. Furthermore, ADAMTS5 is considered one of the most important aggrecan-degrading enzymes in arthritis [11,12] and may also promote lipoprotein binding in atherosclerosis [13].

ECM remodeling is crucial to many developmental and disease processes, in part due to its role in controlling cell signaling. Heparan sulphate proteoglycans bind fibroblast growth factors (FGFs), thereby regulating their bioavailability to their receptors (FGFRs) [14] during developmental processes such as myogenesis [15], as well as acting as co-receptors for Sonic Hedgehog (Shh) signaling [16]. A recent study identified *Adamts9* as necessary for umbilical cord vascular development due, at least in part, on its facilitation of Shh signaling [17]. Furthermore, levels of Hedgehog (Hh) signaling correlate with the severity of osteoarthritis, which is potentially mediated by a pathway involving ADAMTS5 [18]. Combined, these studies are suggestive of a complex interplay between the ECM and crucial cell signaling pathways that involves ADAMTS proteoglycanases.

This study identifies a role for ADAMTS5 during zebrafish embryogenesis. Abrogation of *adamts5* expression disrupted Shh signaling during somite differentiation and reduced the expression of the myogenic regulator *myod*. Importantly, somite differentiation was synergistically dependent upon Shh and ADAMTS5. Moreover, these functions of ADAMTS5 were independent of catalytic function. These data indicate that ADAMTS5 plays an important non-enzymatic role in regulating the Shh pathway during embryogenesis that impacts on muscle development. This may be relevant in conditions where ADAMTS proteins interact with the Shh signaling pathway, such as osteoarthritis and umbilical cord vascular complications, as well as disorders where the myogenic program is disrupted, such as muscular dystrophies. 

## 2. Results

### 2.1. The Secreted Metalloproteinase ADAMTS5 Is Expressed in Zebrafish Embryos

**Figure 1:**
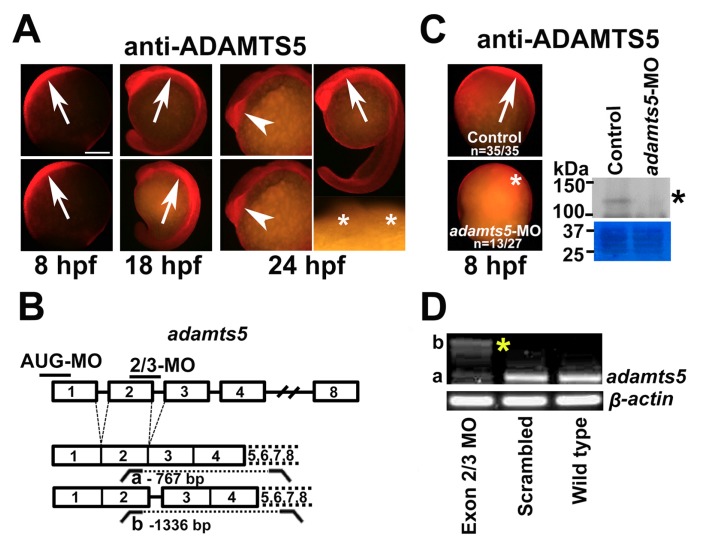
Expression and silencing of *adamts5* in zebrafish embryos. (**A**) ADAMTS5 expression in 8, 18 and 24 hpf wild-type embryos. Note strong early expression in the dorsal mesoendoderm (8 hpf, arrows) and variable expression ventrally (8 hpf, arrowhead), with later expression in the floor plate of the neural tube (18 and 24 hpf, arrows) and bilaterally in the prosencephalon (24 hpf, arrowheads). Asterisks = prosencephalon in no primary antibody control. Scale bar = 250 μm; (**B**) Schematic representation of the *adamts5* gene structure targeted with antisense morpholino oligonucleotides (MO), and its subsequent splicing, indicating the primers used for RT-PCR and the size of the resultant products; (**C**) Reduced ADAMTS5 expression is seen in *adamts5* AUG-MO injected embryos (asterisk) versus control (arrow) by whole-mount antibody labelling (left-hand panel) and Western blot (right-hand panel) showing the 120 kDa ADAMTS5 species (asterisk) with a region of the Coomassie blue stained gel shown below, demonstrating even loading; (**D**) RT-PCR of *adamts5* mRNA obtained from 24 hpf embryos following injection of the *adamts5* 2/3-MO at the 1-cell stage, showing amplicons a and b (asterisk). *β-actin* was used as a house-keeping gene.

We have previously elucidated a role for ADAMTS5 during myoblast fusion in post-natal skeletal muscle from *Adamts5*^−/−^ mice [19]. To investigate this further, zebrafish was employed as a highly manipulable model of vertebrate development, which possesses a strongly conserved *adamts5* gene that is maternally inherited and then dynamically expressed in early-stage embryos [20]. To obtain a detailed understanding of ADAMTS5 protein expression in zebrafish, whole-mount immunohistochemistry (IHC) was performed with a previously described anti-ADAMTS5 antibody directed to its pro-domain [21], which is highly conserved in ADAMTS5 across vertebrates [20,22]. At 8 h post fertilization (hpf) (~80% epiboly), ADAMTS5 was strongly expressed in the dorsal mesoendoderm at the animal pole with variable expression ventrally at the vegetal pole (Figure 1A). At 18 and 24 hpf, after the commencement of somitogenesis, ADAMTS5 was expressed in the rostral neural tube (floor plate) and bilaterally in the prosencephalon (Figure 1A).

### 2.2. Silencing of ADAMTS5 Expression

To explore *adamts5* function, the gene was targeted using two independent morpholino antisense oligonucleotides (MOs) that were directed to either the AUG translation start site (AUG-MO) or the splice site at the exon 2/3 boundary (2/3-MO) (Figure 1B), since exon 3 encodes for the catalytic domain of ADAMTS5 in human, mouse and zebrafish [22]. ADAMTS5 protein expression was found to be reduced upon *adamts5* AUG-MO injection as shown by IHC and immunoblotting (Figure 1C). To confirm altered splicing of *adamts5* transcripts after administration of the 2/3-MO, RT-PCR was performed followed by sequencing analysis (Figure 1D). This indicated a 71% reduction of correctly spliced *adamts5* transcript and identified an alternate *adamts5* transcript retaining the 569-bp intron between exons 2 and 3 that results in inclusion of several premature stop codons (Figure 1D). The AUG-MO was subsequently used throughout the study to ensure translation of the entire gene was disrupted, as well as to guarantee the maternal transcripts for this gene [20] were also affected; however, similar data was obtained with the *adamts5* 2/3-MO [23]. 

### 2.3. Notochord Morphology Is Perturbed in adamts5 Morphant Embryos

**Figure 2:**
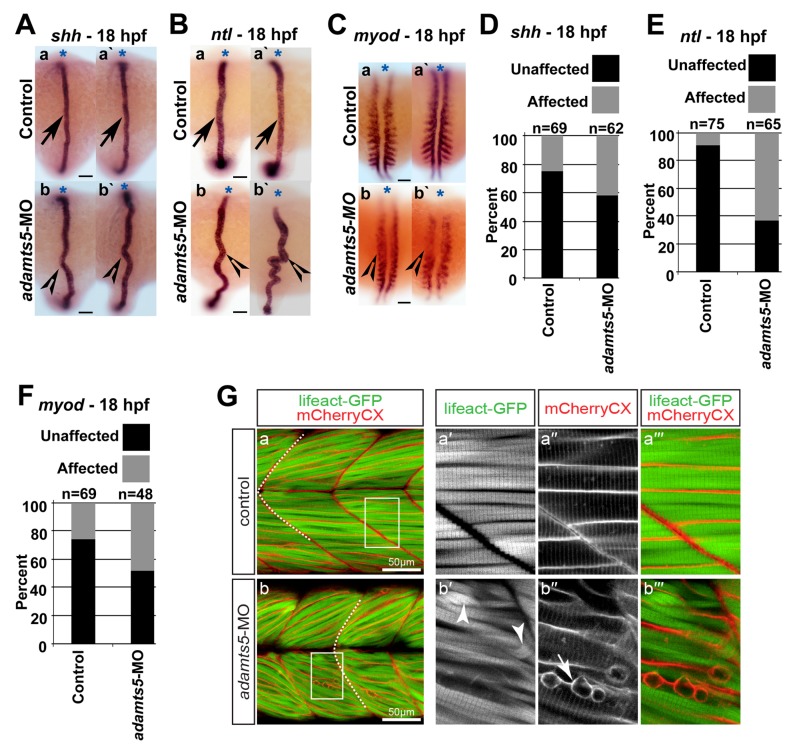
Notochord morphogenesis and muscle fiber formation is perturbed in *adamts5* morphant embryos. (**A**) Expression of *shh* in the notochord of 18 hpf control (**a**,**a′**, arrows) and *adamts5* morphant (**b**,**b′**, open arrowheads) embryos, with medio-lateral deviation in the *adamts5* morphants (**b**,**b′**), with anterior indicated (*); (**B**) Expression of *ntl* in the notochord of 18 hpf control (**a**,**a′**, arrows) and *adamts5* morphant (**b**,**b′**, open arrowheads) embryos, with medio-lateral deviation in the *adamts5* morphants with anterior indicated (*); (**C**) Expression of *myod* in adaxial and paraxial mesoderm of 18 hpf control embryos (**a,a′**) and its perturbation in *adamts5* morphants (**b**,**b′**, open arrowheads) with anterior indicated (*); (**D**) Quantitation of affected notochords in control and *adamts5* morphant embryos demarcated by *shh* in Figure 2A; (**E**) Quantitation of affected notochords in control and *adamts5* morphant embryos demarcated by *ntl* in Figure 2B; (**F**) Quantitation of embryos with perturbed *myod* expression in control and *adamts5* morphant embryos demarcated in Figure 2C; (**G**) Double-transgenic *Tg(acta1:lifeact-GFP)*/*Tg(acta1:mCherryCaaX)* embryos, in which thin filaments are marked green and sarcolemma red, reveal loss of muscle integrity in 3 dpf *adamts5* morphants. Muscle fibers of control injected larvae feature the typical striation of the myofibril and regular myofibers within chevron-shape somites, indicated by a dashed line (**a**). The boxed area in **a** is magnified in **a′**–**a′′′**. Myofibril striation is partially lost within *adamts5* morphants (arrowhead in **b′**) and the sarcolemma of the myofibers disrupted (arrow in **b′′**). The boxed area in **b** is magnified in **b′**–**b′′′**. Scale bar = 50 μm.

Shh signaling from the notochord has been previously demonstrated to be important for adaxial and paraxial mesoderm formation and *myod* expression during myogenesis [24], while *no tail* (*ntl*) is an independent marker for axial mesoderm (notochord) [25]. Expression of *shh* and *ntl* remained unchanged in 12 hpf *adamts5* morphants compared to controls [23]. However, at 18 hpf the pattern of *shh* (Figure 2A,D) and *ntl* (Figure 2B,E) staining was altered revealing disrupted notochord morphology. 

### 2.4. Skeletal Muscle Formation Is Disrupted in adamts5 Morphant Embryos

Notochord perturbation is linked with defective somitic muscle formation and morphogenesis [24]. Therefore, the disrupted notochord morphology in the *adamts5* morphants suggested that skeletal muscle development might be affected. This is also consistent with previous observations indicating a skeletal muscle developmental defect in *Adamts5* knockout mice [19]. Reduced or absent paraxial mesodermal *myod* expression was also observed at 18 hpf (Figure 2C,F). To analyze potential myofiber defects, *adamts5* AUG-MO was administered to double-transgenic embryos, in which myofiber thin filaments were labeled with Lifeact-GFP whereas the sarcolemma and t-tubules of the myofiber were marked with mCherryCaaX via the CaaX-tag [26]. In control injected 3 dpf double-transgenic larvae, Lifeact-GFP revealed the typical striation of the highly organized myofibril and mCherryCaaX indicated regularly spaced t-tubules and ordered fiber membranes within chevron-shaped somites (Figure 2Ga–a′′′). In contrast, the somites of *adamts5* morphants were U-shaped, which resembled a phenotype previously reported in *shh* mutant embryos [27] (Figure 2G(b)), confirming *shh* availability as a potential cause. In addition, myofibril striation within myofibers of *adamts5* morphants was partially lost and the sarcolemma appeared irregular, indicating disrupted muscle organization (Figure 2Gb–b′′′).

**Figure 3:**
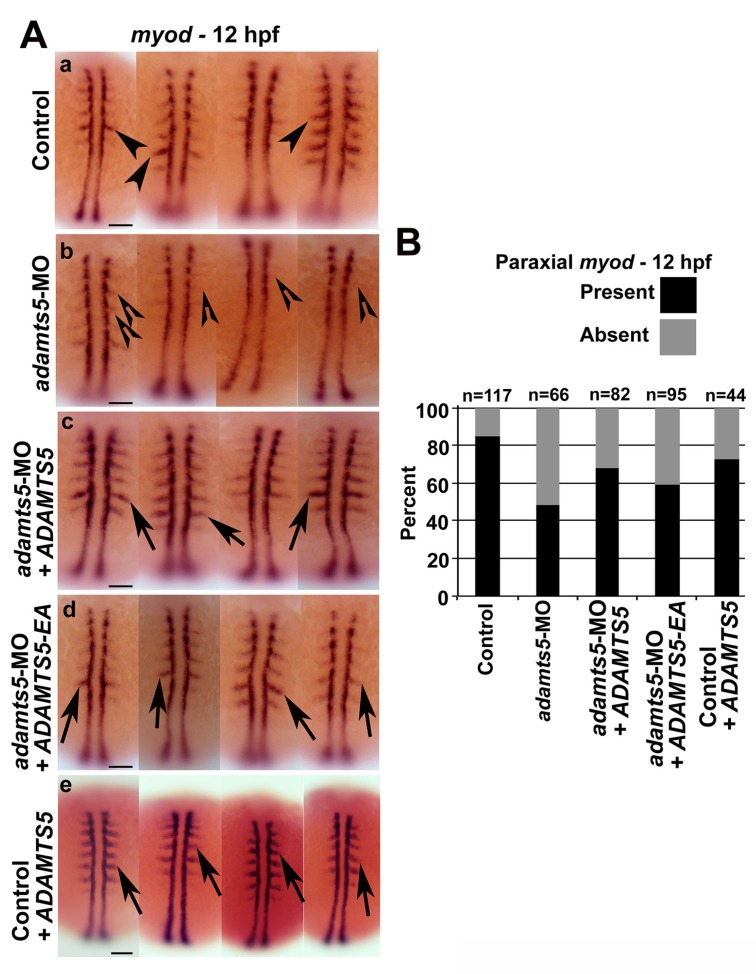
Loss of paraxial mesodermal *myod* expression in *adamts5* morphant embryos. (**A**) Expression of adaxial and paraxial *myod* in 12 hpf embryos injected with control MO (**a**, arrowheads), with *adamts5* morphant embryos showing substantial loss of paraxial expression (**b**, open arrowheads), as well as mild loss of paraxial *myod* expression (**b**, open arrowheads). Rescue of paraxial *myod* expression in *adamts5* morphants co-injected with mRNA encoding wild-type (**c**, arrows) or catalytically-inactive E^411^A (**d**, arrows) ADAMTS5. Control embryos injected with *ADAMTS5* mRNA encoding wild-type ADAMTS5 show unaffected *myod* expression in paraxial mesoderm (**e**, arrows). Scale bar = 100 μm; (**B**) Quantitation of embryos showing present or absent *myod* patterning represented in (**A**).

To further analyze myofiber differentiation, *myod* expression was examined. Reduced or absent paraxial mesodermal *myod* expression was observed at 12 hpf (Figure 3A(a,b)), whereas expression of adaxial mesodermal *myod* was largely unaffected (Figure 3A(a,b)). Similar observations were made with the *adamts5* 2/3-MO (Supplementary Figure S1) or upon co-injection of a *p53* morpholino with the *adamts5* AUG-MO (Supplementary Figure S2B). To ensure that the specificity of the phenotype was due to reduced *adamts5* expression, mRNA encoding either wild-type or catalytically-inactive (E^411^A) ADAMTS5 were co-injected, with both able to partially rescue the reduced paraxial mesodermal *myod* expression (Figure 3A(c,d), respectively, and Figure 3B). This indicated that the enzymatic function of ADAMTS5 was not necessary to induce the reduced *myod* expression.

### 2.5. Receptor-Mediated Sonic Hedgehog Signaling Is Affected in adamts5 Morphants

**Figure 4:**
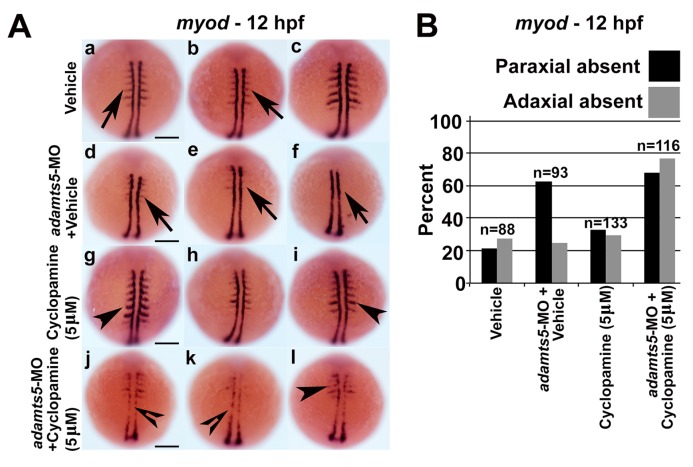
Combinatorial inhibition of Shh signaling and *adamts5* disrupt paraxial and adaxial *myod* expression. (**A**) Adaxial and paraxial *myod* expression in 12 hpf embryos treated with vehicle control (**a**–**c**, arrows denote paraxial *myod* expression), vehicle + *adamts5*-MO (**d**–**f**, arrows denote absent paraxial *myod* expression), 5 μM cyclopamine (**g**–**i**, arrowheads represent similar paraxial *myod* staining compared to control group) and 5 μM cyclopamine + *adamts5*-MO (**j**–**l**, open arrowheads represent absent adaxial *myod* expression and arrowhead represents absent paraxial *myod* staining compared to *adamts5* MO group). Scale bar = 200 μm; (**B**) Quantitation of embryos showing present or absent *myod* patterning represented in (**A**).

**Figure 5:**
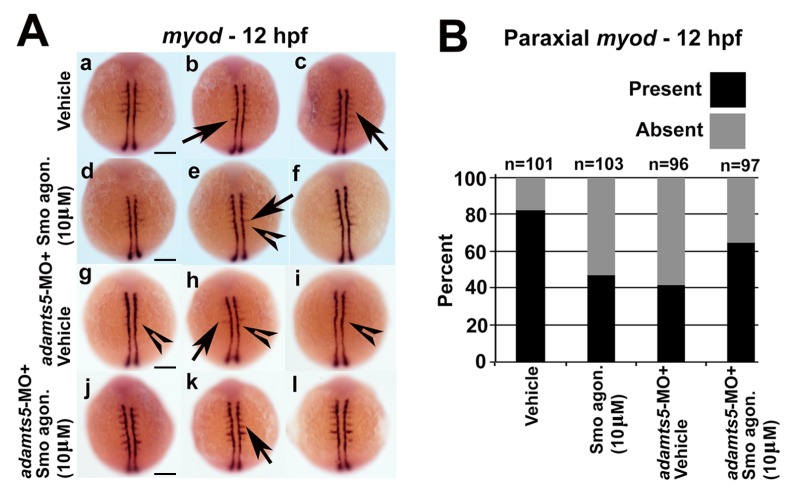
Combinatorial activation of Shh signaling and inhibition of *adamts5* rescues paraxial *myod* expression. (**A**) Adaxial and paraxial *myod* expression in 12 hpf embryos treated with vehicle control (**a**–**c**, arrows), 10 μM Smoothened agonist (Smo agon.) (**d**–**f**, arrow/open arrowhead represent present/absent *myod* expression in paraxial mesoderm), vehicle + *adamts5*-MO (**g**–**i**, arrow/open arrowhead represents present/absent *myod* expression in paraxial mesoderm) and 10 μM Smo agon. + *adamts5*-MO (**j**–**l**, arrows indicate *myod* expression present in paraxial mesoderm). Scale bar = 100 μm; (**B**) Quantitation of embryos showing present or absent *myod* patterning represented in (**A**).

**Figure 6:**
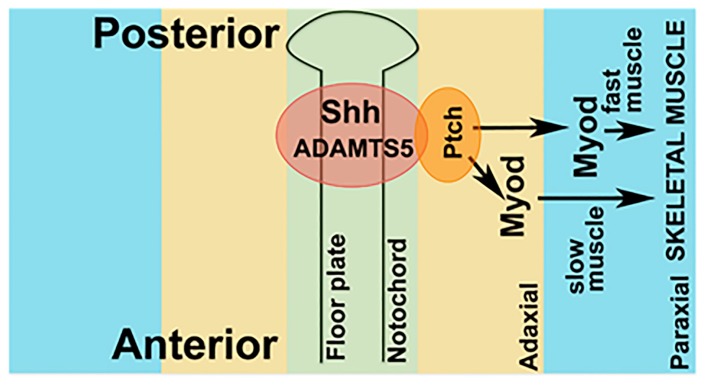
Model of interaction between Shh and ADAMTS5. Hypothetical model showing ADAMTS5 and Shh expression synergistically regulate downstream activation of MyoD in adaxial and paraxial mesoderm.

We hypothesized that reduced ADAMTS5 could lead to an altered extracellular environment that might disrupt Shh signaling, and that since adaxial mesoderm is in closer proximity to the notochord it might be less disrupted compared to the paraxial mesoderm. Therefore, cyclopamine, an antagonist of Smoothened (Smo), a receptor in the Shh signaling pathway [28] was used to understand whether Shh signaling through Smo was impaired in *adamts5* morphants. The presence of 5 μM cyclopamine did not affect adaxial *myod* expression at 12 hpf in wild-type embryos (Figure 4A(g–I),B). However, treatment of *adamts5* morphants with 5 μM cyclopamine severely affected adaxial expression of *myod* (Figure 4A(j–l),B) compared to untreated *adamts5* morphant embryos (Figure 4A(d–f),B). In a reciprocal experiment, the Smo agonist, SAG, was used to confirm the dependency of Shh signaling on *adamts5* expression. Administration of SAG on wild-type embryos disrupted paraxial *myod* expression in a similar manner to *adamts5* morphants (Figure 5A(d–I),B). However, the same concentration of SAG partially rescued the loss of paraxial *myod* patterning in the *adamts5* morphants (Figure 5A(g–l),B). These experiments collectively suggest an interaction between ADAMTS5 and Shh, such that they act synergistically to stimulate *myod* expression in adaxial mesoderm (Figure 6).

## 3. Discussion

Zebrafish myogenesis is controlled by multiple pathways [29,30,31]. Signaling from the notochord specifies slow-twitch muscle precursors in the adaxial mesoderm [24], which migrate laterally after somite formation to the most lateral muscle layer [32]. Myotomes develop following elongation and fusion of somitic cells and their attachment to the somite boundary, with the boundaries between myotome forming the critical myotendenous junctions that are the primary sites of force generation [33]. ECM–cell adhesion has been shown to be essential for multiple steps in this process [34]. This study has identified a novel non-catalytic function of the ECM protein ADAMTS5 in regulating Sonic Hedgehog signaling that impacted on somite differentiation, with reduced expression of *myod* in the paraxial mesoderm and disrupted myotome boundaries.

The phenotypes induced by the *adamts5* morphants were rescued with mRNA encoding both wild-type and catalytically-inactive ADAMTS5. Although unexpected, there is some precedence for ADAMTS family members demonstrating non-catalytic functions as reviewed recently [35]. For example, ADAMTS1 has been shown to bind to VEGF through its C-terminal thrombospondin repeats and spacer domain to block VEGFR2 activation [5]. Moreover, both wild-type and catalytically-inactive (E^363^A) ADAMTS15 were able to reduce breast cancer cell migration on matrices of fibronectin or laminin [6]. Furthermore, enzymatic activity was not required for enhancement of neurite outgrowth by ADAMTS4, which was instead dependent upon MAP kinase cascade activation [7]. ADAMTSL family members, which are structurally similar to ADAMTS family members but lack the N-terminal propeptide and catalytic domain, may also offer some important insights into non-catalytic functions of ADAMTS family members. Most notably, mutations of human ADAMTSL2 have been causally linked to the musculoskeletal disorder Geleophysic Dysplasia [36], where patients present with severe short stature, joint immobility and cardiac valvular abnormalities. Collectively, this suggests a role of ADAMTS5 in zebrafish muscle development is likely not related to its enzymatic function. However, mouse studies have highlighted considerable redundancy amongst ADAMTS members [35] suggesting that combinatorial targeting might be required to identify additional functions that may be dependent on enzymatic activity.

Shh is an important regulator of musculoskeletal development, given its role in somite and neural tube patterning. Duplication, and presumed overexpression, of Shh is associated with congenital muscular hypertrophy in humans [37]. Shh also enables the formation of the cranial musculature [38] and polarizes the limb during early morphogenesis [39,40]. Shh has also been demonstrated to mediate the patterning of somites [41,42]. Shh has the ability to activate myogenesis in vitro and in vivo [43] with expression and secretion of Shh from the notochord able to induce slow muscle fiber formation in vivo via *myod* [24]. The *adamts5* morphants displayed altered *myod* expression in the paraxial—but not adaxial—mesoderm despite levels of *shh* expression in the notochord being unaffected. This might be explained by reduced bioavailability of Shh in the absence of ADAMTS5. Since the adaxial *myod*-positive cells represent the slow muscle precursors that subsequently move through the fast muscle region where they impact on fast muscle differentiation [44], it would be of interest to examine the relative distribution of slow and fast twitch muscle fibers in the *adamts5* morphants.

The results obtained using agonists and antagonists of the downstream Smo pathway suggest that ADAMTS may work both upstream, as well as in parallel with Shh signals. Wnt/β-catenin signaling has been shown to act in co-operation with Shh (and BMPs) in embryonic myogenesis [31]. This could be mediated, at least partially, via ADAMTS5 since Wnt/β-catenin has been shown to act upstream of ADAMTS5 in other developmental situations, such as chondrocyte maturation and function [45]. Similarly, defects in Delta/Notch can affect somite boundary formation [46], with this pathway also shown to induce ADAMTS5 in joint cartilage, providing another potential upstream regulator of ADAMTS5 during somite differentiation.

Defective notochords have been identified in mutants of ECM components, such as fibrillin [47], collagen [48], the basement membrane proteins laminin alpha [49], beta and gamma [50], as well cell-associated molecules such as integrins [46]. A number of these defects are due to disrupted morphogenesis that results from perturbed ECM-cell interactions [49]. This suggests that altered morphogenesis as well as disrupted patterning may contribute to the perturbed notochord in *adamts5* morphants. In addition, U-shaped myotome boundaries have also been observed in mutants of ECM components, such as fibronectin [51] and laminin [52], or the alternative ECM processing enzyme MMP-11 [53], providing precedence for ADAMTS5 impacting on the myotome boundary.

This study has identified a new function for the metzincin ADAMTS5. By exploring the role of ADAMTS5 in zebrafish, understanding has been gained of a potential non-catalytic function in the regulation of muscle development and maintenance via interaction with the Sonic Hedgehog signaling pathway. Since both ADAMTS5 and Shh have independent—as well as potential combinatorial—roles during musculoskeletal development, the complex interplay between ADAMTS and Shh could be relevant to the development of musculoskeletal diseases, such as muscular dystrophies and arthritis. Further biochemical and functional characterization of potential interactions between ADAMTS5 and Shh in such diseases may reveal new insights into the development and progression of these diseases. Given that treatment options for these diseases are limited, this knowledge could then be applied to the development of novel therapeutics that specifically modulate this interaction to slow the progression of these debilitating conditions.

## 4. Materials and Methods

### 4.1. Zebrafish Lines and Maintenance

Wild-type and *Tg(acta1*:*lifeact-GFP)/Tg(acta1:mCherryCaax)* [26] zebrafish were maintained, raised and staged according to standard protocols [54]. Embryos were obtained by mating trios or using a mass embryo production system (MEPS) (Aquatic Habitats) and raised at 28.5 °C. Experiments were approved by the Deakin University Animal Ethics Committees (G14/2013, 15/05/2013). 

### 4.2. Embryo Microinjection and Other Treatments

Morpholino antisense oligonucleotides (MOs; Gene Tools) targeting the ATG start codon (5′-atgctgtcgaaattacaggagtttggcgcgtat) and exon 2/3 splice site (5′-ctatcattgaggacgacggcctgcacgctgccttcactgtggctcatgagatc) of zebrafish *adamts5* (GenBank: JF778846.1) were used to ablate the *adamts5* gene. MOs were solubilized in 1× Danieau buffer and 1 nL injected at a concentration of 1 mg/mL into one-cell stage embryos, as previously described [55]. Alternatively-spliced *adamts5* species were confirmed by Sanger sequencing (Australian Genome Research Facility, Melbourne, Australia) of RT-PCR amplicons generated with flanking primers (5′-ggcggatgtaggaactgtgt and 5′-ttacgcacctcacactgctc). Capped RNA encoding full-length wild-type or catalytically-inactive (E^411^A) ADAMTS5 [21] were synthesized using the T7 mMessage mMachine kit (Thermofisher Scientific, Scoresby, Victoria, Australia) and 40 pg was microinjected into one-cell stage embryos.

For other studies, injected embryos were treated with 5 µM cyclopamine (Sigma-Aldrich, St. Louis, MO, USA) in DMSO or 10 µM Smoothened agonist SAG (CAS 364590-63-6) (Merck Millipore, Darmstadt, Germany) in water, along with the corresponding vehicle control at 5.5 hpf and fixed at 12 hpf in 4% PFA/PBS.

### 4.3. Whole-Mount In Situ Hybridization and Immunofluorescence

Whole-mount in situ hybridization was performed as described [56]. The following antisense digoxigenin-labelled mRNA probes were synthesized by in vitro transcription: *shha* and *myod* [57], and *no-tail* (*ntl*) [58]. Immunofluorescence performed on whole embryos with polyclonal rabbit anti-propeptide ADAMTS5 (Cat# ab39203-100, Abcam, Pak Shak Kok, New Territories, Hong Kong, China) at 1:200 followed by anti-rabbit Alexa fluor 594 secondary antibody at 1:500 (Life Technologies, Carlsbad, CA, USA). Histochemical methods were performed as previously described [59].

### 4.4. Western Blotting

Cell lysates were extracted from 24 hpf embryos and subjected to Western blotting as described previously [60], using the polyclonal rabbit antibody against propeptide ADAMTS5 described above at 1:5000 followed by an anti-rabbit HRP antibody (Cell Signaling Technologies, Danvers, MA, USA) at 1:10,000. Protein concentrations were measured using the Bradford assay and equal loading of protein confirmed by Coomassie blue staining on duplicate SDS-PAGE gels. 

### 4.5. Statistics

Two-tailed paired *t*-tests were performed between all treatment groups compared to the respective control groups with scoring performed blind. Significance was achieved at a *p*-value ≤ 0.05, with Gaussian distribution assumed in all cases.

## Supplementary Materials

Supplementary materials can be found at http://www.mdpi.com/1422-0067/19/3/766/s1.
