# Supplementary material for: The ADAMTS5 Metzincin Regulates Zebrafish Somite Differentiation
Source: Int J Mol Sci. 2018 Mar 7;19(3):766. doi: 10.3390/ijms19030766 (PMC5877627; doi:10.3390/ijms19030766)
Supplement: Supplementary file 1 [file ijms-19-00766-s001.pdf]

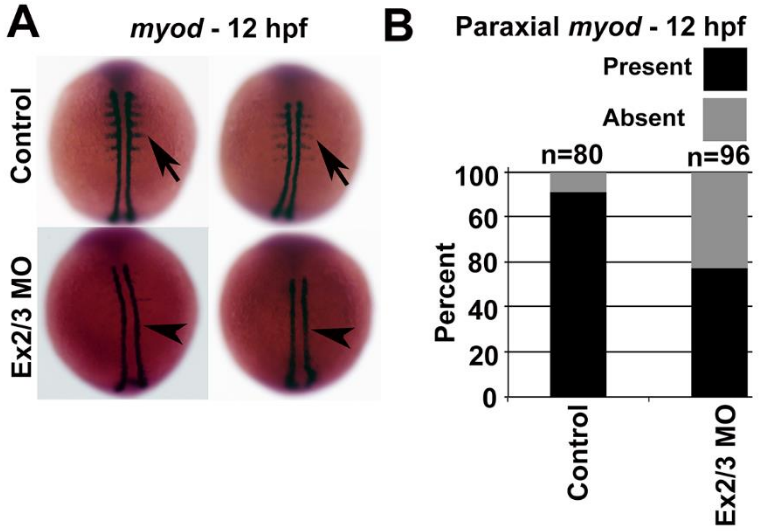

Supplementary Figure 1. Expression of *myod* is altered in ADAMTS5 morphant embryos. (A) Expression of *myod* in paraxial mesoderm of 12 hpf control (arrows) and *adamts5* exon 2/3 MO injected embryos. Note perturbation of expression in the *adamts5* morphants (arrowheads). (B) Percent of embryos with perturbed *myod* expression in control and *adamts5* morphant embryos represented in Supp Fig 1A.

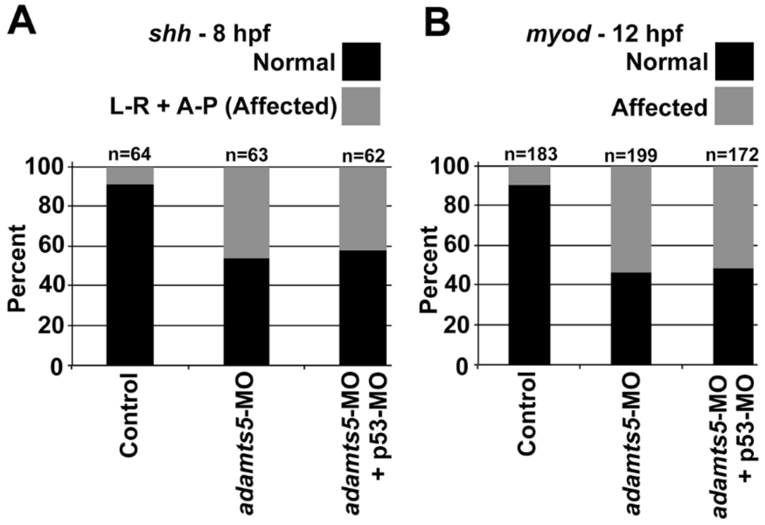

Supplementary Figure 2. Quantitation of *shh* and *myod* disruption in ADAMTS5 morphant embryos. (A) Percent of embryos showing normal or disrupted *shh* patterning in control, *adamts5*-MO or *adamts5*-MO + P53-MO injected embryos. (B) Percent of embryos showing normal or affected *myod* patterning in control, *adamts5*-MO or *adamts5*-MO + P53-MO injected embryos.
